# An unusual aspect of Pott’s disease: small bowel obstruction from a tuberculous psoas abscess

**DOI:** 10.1093/jscr/rjag023

**Published:** 2026-02-05

**Authors:** Bikram Bhandari, Suresh Prasad Shah, Dinesh Nalbo, Kabita Neupane, Grishma Khadka, Aashish Baniya

**Affiliations:** B.P. Koirala Institute Of Health Sciences, Dharan 56700, Nepal; Department of Surgery, B.P. Koirala Institute of Health Sciences, Dharan 56700, Nepal; Department of Surgery, Birat Medical College Teaching Hospital, Biratnagar 56613, Nepal; Department of Radiodiagnosis and Imaging, B.P. Koirala Institute of Health Sciences, Dharan 56700, Nepal; Department of Surgery, B.P. Koirala Institute of Health Sciences, Dharan 56700, Nepal; B.P. Koirala Institute Of Health Sciences, Dharan 56700, Nepal

**Keywords:** Pott’s disease, spinal tuberculosis, psoas abscess, small bowel obstruction, ileal thickening

## Abstract

Pott's disease typically presents with back pain and systemic symptoms, but atypical presentations can obscure diagnosis. We describe a rare manifestation where the initial presentation was acute small bowel obstruction. A 20-year-old female presented with acute abdominal pain, distension, and vomiting, with a 3-month history of intermittent lower back pain. Computed tomography scan revealed distal ileal obstruction due to wall thickening adjacent to a left psoas abscess and a large paravertebral abscess. Aspirated pus confirmed *Mycobacterium tuberculosis*. The diagnosis was small bowel obstruction secondary to ileal thickening from an adjacent psoas and paravertebral abscesses. She was managed conservatively with abscess drainage and anti-tuberculous therapy. Intestinal obstruction is a rare presentation of spinal tuberculosis. In endemic areas, tuberculosis should be considered in the differential diagnosis of acute abdomen. Prompt imaging and microbiological confirmation, coupled with multidisciplinary management, are essential to achieve favorable outcomes.

## Introduction

Tuberculosis of the spine, also known as Pott’s disease, is one of the oldest diseases known to humans [1]. It is the most severe and commonly encountered extrapulmonary type of tuberculosis [2]. Spinal TB accounts for almost 50% of all osteoarticular TB cases, and 1%–3% of all TB cases [3]. The burden of musculoskeletal TB, particularly spinal involvement, remains disproportionately high in developing countries, accounting for significant morbidity [4].

Spinal TB commonly affects the thoracolumbar segments and typically involves the vertebral body and intervertebral space, but rarely affects only the adnexa [3]. Pott’s spine can cause vertebral destruction, collapse, kyphotic deformity, and the formation of paravertebral cold abscesses [5]. The psoas muscle is a common conduit, with abscesses frequently spreading along its sheath, leading to psoas abscess formation [6, 7].

Patients usually present with chronic back or flank pain, fever, weight loss, and night sweats, but symptoms may be vague and misleading [6]. In rare cases, presentation may mimic acute abdomen, with bowel obstruction or fistula formation due to abscess extension involving the retroperitoneum and bowel wall [2, 8].

This case describes an unusual presentation of Pott’s disease where the initial manifestation was acute small bowel obstruction caused by inflammatory involvement of the ileum from an adjacent psoas and paravertebral abscess.

## Case report

A 20-year-old female presented with a one-day history of severe, continuous, non-radiating upper abdominal pain, worsened by food intake, associated with multiple episodes of non-bilious, non-projectile vomiting along with abdominal distension which initially involved the central abdomen then progressed to whole abdomen, and inability to pass stool or flatus. She also reported three months history of intermittent dull left-sided low back pain, aggravated by prolonged standing and movement, and relieved with analgesics.

On examination, she was afebrile and hemodynamically stable. The abdomen was distended with mild epigastric and right inguinal region tenderness without guarding or rebound tenderness. On auscultation, bowel sounds were sluggish. Neurological examination of the lower limbs was normal.

### Investigations

Complete blood count showed leukocytosis (11 700/mm^3^), erythrocyte sedimentation rate 55 mm/hr, and C-reactive protein 48 mg/L. Renal and liver function tests were within normal limits. HIV, HBsAg, and hepatitis C virus were non-reactive.

**Figure 1:**
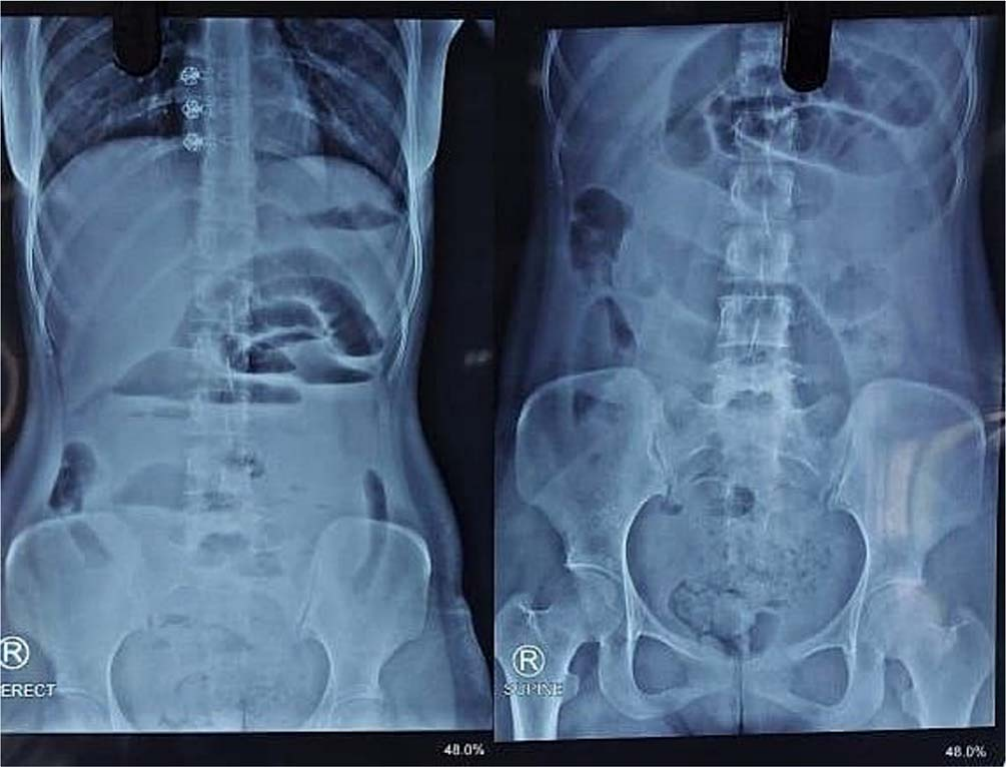
X-ray abdomen showing dilated small bowel loops with faecal loading (erect and supine).

**Figure 2:**
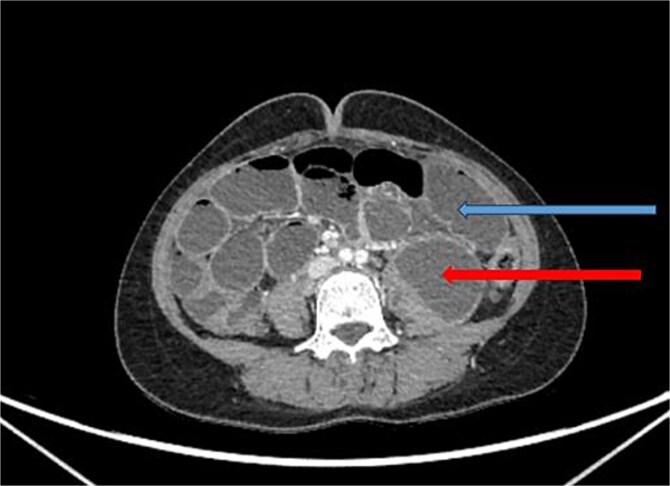
CT abdomen/pelvis with contrast, upper arrow showing dilated small bowel loops and lower arrow showing collection in left psoas muscle.

**Figure 3:**
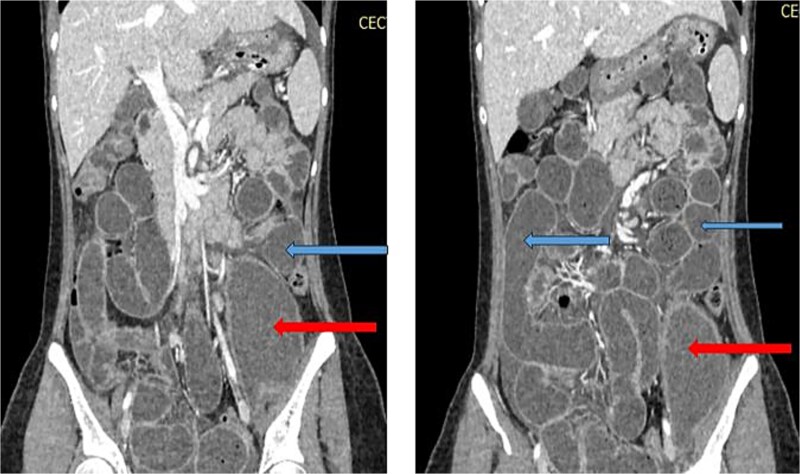
CT abdomen/pelvis with contrast, upper arrows showing dilated small bowel loops and lower arrrows showing collection in left psoas muscle.

X-ray abdomen showed dilated small bowel loops with faecal loading (Fig. 1). Ultrasound abdomen/pelvis revealed dilated small bowel loops with to-and-fro peristalsis, suggestive of small bowel obstruction. Contrast-enhanced computed tomography (CT) abdomen/pelvis revealed distal ileal thickening causing obstruction, a left iliopsoas abscess communicating with a paravertebral abscess (L5–S2), and erosion of the L5 vertebral body (Figs 2 and 3).

### Microbiological confirmation

GeneXpert Ultra on aspirated pus confirmed *Mycobacterium tuberculosis* with low bacillary load with no rifampicin resistance.

The final diagnosis was acute small bowel obstruction due to left iliopsoas and paravertebral abscess secondary to Pott’s disease.

### Management

**Figure 4:**
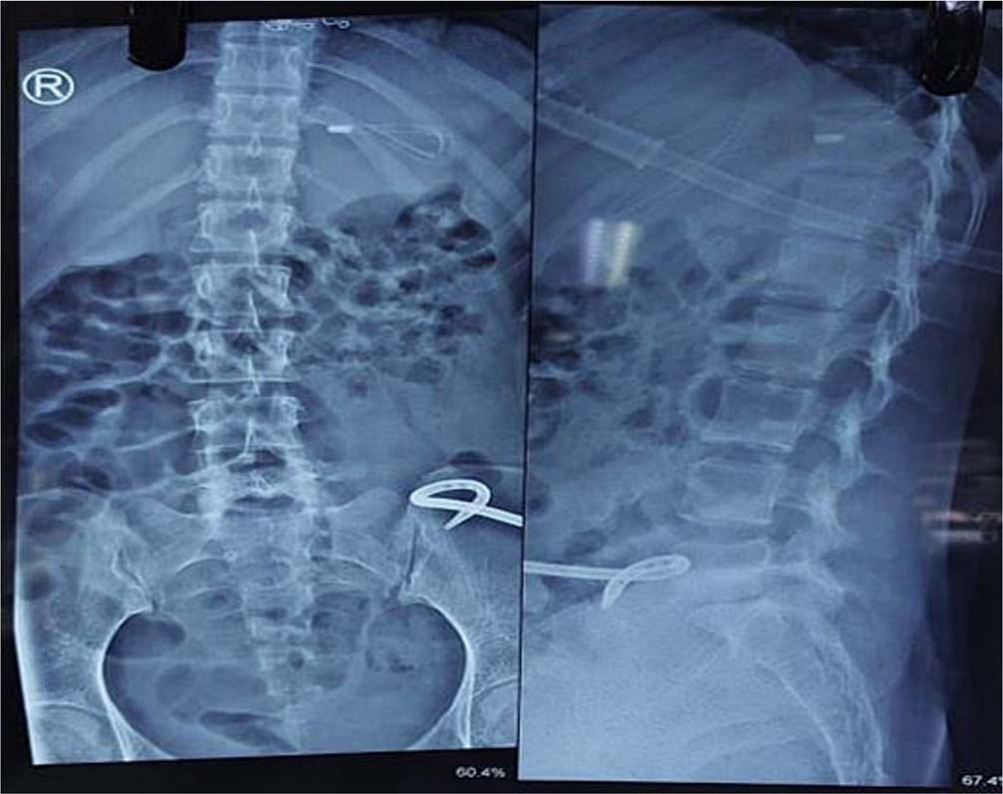
X-ray abdomen showing pig tail catheter in left inguinal region and nasogastric tube in stomach (anteroposterior and lateral).

She was treated conservatively with IV fluids, analgesics, and antibiotics. Ultrasound-guided catheter drainage of the abscess yielded ~100 ml of pus. A post-procedure X-ray confirmed the position of the drainage catheter (Fig. 4). Anti-tuberculous therapy (ATT) was initiated under the DOTS strategy. Her obstruction resolved, and she was discharged on Day 4 with advice to continue ATT as per DOTS, a high-protein diet and follow-up in the surgery outpatient department. Orthopaedics consultation advised lumbar corset for ambulation support.

## Discussion

This case demonstrates an unusual clinical manifestation of spinal tuberculosis, in which the dominant symptoms were those of an acute abdominal emergency i.e. pain abdomen, vomiting and abdominal distension, effectively masking the primary spinal source. The diagnostic process in this patient highlights the difficulties in identifying Pott's disease when it presents with non-classical, vague systemic complications, a challenge often encountered in clinical practice [9]. Correct diagnosis required correlating the chronic back pain with radiological findings.

Malhotra *et al.* [1] identified medical imaging as fundamental for the assessment of Pott’s disease. Magnetic resonance imaging (MRI) is often considered the gold standard for early diagnosis due to superior soft tissue resolution and detection of marrow edema, abscesses, and neural compression [10]. In our patient, CT provided critical diagnostic detail, showing bone destruction, abscess extension, and bowel involvement.

This unusual presentation has been rarely reported. Chepuri [2] described a case initially mistaken for intestinal obstruction before spinal TB was confirmed. Saint Clair *et al.* [6] reported Pott’s disease with psoas abscess, while Hodgson & Yau [7] historically documented the psoas muscle as a common abscess pathway. Srivastava *et al.* [5] detailed surgical management of extensive abscesses. A summary of similar published cases is provided in Table 1.

**Table 1:** Published cases of spinal tuberculosis with unusual abdominal presentations

Author/Year	Age/Sex	Presentation	Imaging findings	Final diagnosis	Management	Outcome
Chepuri (2021) [2]	26/M	Acute intestinal obstruction	CT: dilated bowel loops; spinal changes detected later	Spinal TB misdiagnosed initially as intestinal obstruction	ATT + supportive care	Resolved
Saint Clair *et al.* (2022) [6]	45/M	Back pain, fever, psoas abscess	CT: psoas abscess with vertebral involvement	Pott’s disease with psoas abscess	Surgical drainage + ATT	Good recovery
Hodgson & Yau (1967) [7]	Series	Back pain, cold abscess	Imaging (plain X-ray era): spinal destruction with abscess	Psoas abscess complicating spinal TB	Open surgical drainage + ATT	Improved
Srivastava *et al.* (2017) [5]	30s–40s (series)	Extensive abscess with neuro deficits	MRI/CT: large multiloculated spinal abscesses	Spinal TB with extensive collections	Surgical decompression + ATT	Improved
Present Case (2025)	20/F	Acute abdomen with small bowel obstruction	CT: distal ileal thickening, paravertebral & psoas abscess, L5 erosion	Small bowel obstruction secondary to Pott’s disease	Percutaneous drainage + ATT	Full recovery

Management of such complex cases is increasingly based on minimally invasive strategies. Drainage of abscesses combined with prompt ATT is effective in most patients [3, 5], avoiding major surgery unless neurological compromise or instability develops. WHO guidelines emphasize early multi-drug ATT to achieve cure [8]. This case is unique in that small bowel obstruction was the primary presentation, caused by direct bowel wall involvement from tuberculous inflammation, a phenomenon rarely documented in the literature.

## Conclusion

Spinal tuberculosis is notorious for atypical presentations. This report illustrates that intestinal obstruction can be an uncommon yet severe consequence of advanced Pott’s disease, caused by extension of a psoas abscess to the bowel wall. Clinicians in TB-endemic regions should maintain a high index of suspicion when evaluating acute abdomen in young patients with subtle spinal symptoms. Early imaging, microbiological confirmation, and multidisciplinary management—combining abscess drainage with ATT—are key to favorable outcomes.
